# Global financing for health policy and systems research: a review of funding opportunities

**DOI:** 10.1093/heapol/czac109

**Published:** 2022-12-22

**Authors:** Alexander Kentikelenis, Abdul Ghaffar, Martin McKee, Livia Dal Zennaro, David Stuckler

**Affiliations:** Department of Social and Political Sciences, Bocconi University, via Roentgen 1, Milan 20136, Italy; Alliance for Health Policy and Systems Research, World Health Organization, 20 Avenue Appia, Geneva 1211, Switzerland; Department of Health Services Research and Policy, London School of Hygiene and Tropical Medicine, 15-17 Tavistock Place, London WC1H 9SH, UK; Alliance for Health Policy and Systems Research, World Health Organization, 20 Avenue Appia, Geneva 1211, Switzerland; Department of Social and Political Sciences, Bocconi University, via Roentgen 1, Milan 20136, Italy

**Keywords:** Health policy and systems research (HPSR), global health financing, donor priorities, health systems

## Abstract

Health policy and systems research (HPSR) is a neglected area in global health financing. Despite repeated calls for greater investment, it seems that there has been little growth. We analysed trends in reported funding and activity between 2015 and 2021 using a novel real-time source of global health data, the Devex.com database, the world’s largest source of funding opportunities related to international development. We performed a systematic search of the Devex.com database for HPSR-related terms with a focus on low- and middle-income countries. We included ‘programs’, ‘tenders & grants’ and ‘contract awards’, covering all call statuses (open, closed or forecast). Such funding opportunities were included if they were related specifically to HPSR funding or had an HPSR component; pure biomedical funding was excluded. Our findings reveal a relative neglect of HPSR, as only ∼2% of all global health funding calls included a discernible HPSR component. Despite increases in funding calls until 2019, this situation reversed in 2020, likely reflecting the redirection of resources to rapid assessments of the impacts of the coronavirus disease 2019 (COVID-19) pandemic. Most identified projects represented small-scale opportunities—commonly for consultancies or technical assistance. To the extent that new data were generated, these projects were either tied to a specific large intervention or were narrow in scope to meet a specific challenge—with many examples informing policy responses to the Covid-19 pandemic. Nearly half of advertised funding opportunities were multi-country projects, usually addressing global policy priorities like health systems strengthening or development of coordinated public health policies at a regional level. The Covid-19 pandemic has shown why investing in HPSR is more important than ever to enable the delivery of effective health interventions and avoid costly implementation failures. The evidence presented here highlights the need to scale up efforts to convince global health funders to institutionalize the inclusion of HPSR components in all funding calls.

**Key messages**

- This article examines financing available for health policy and systems research (HPSR) by global donors. To do this, it relies on a novel source of data: HPSR-related funding opportunities extracted from the world’s largest database of international development projects, Devex.com.
- The article documents that global donors neglect HPSR in their projects and funding calls. Only ∼2% of all global health funding opportunities included a discernible HPSR component.
- Most HPSR funding opportunities are small-scale opportunities: commonly, short-term consulting projects or technical assistance. Large funding calls (>$1 million) remain primarily focused on biomedical research, neglecting to embed support for HPSR activities within the remit of larger global health projects.
- Available funding calls for HPSR have been decreasing over the past 2 years, despite the coronavirus disease 2019 pandemic highlighting why investing in HPSR is more important than ever to enable the delivery of effective health interventions.

## Introduction

In recent years, both scholars and policymakers have increasingly recognized the need to increase investment in ‘health policy and systems research’ (HPSR) (Sheikh *et al.*, 2011; Shroff *et al.*, 2017; Bennett *et al.*, 2018; Dugle *et al.*, 2020). By HPSR, we refer to research on the policies and systems that can improve health goals, typically through the planning, implementation and evaluation of global health projects (Gilson, 2012). Yet, despite repeated calls to increase HPSR allocations, there is limited evidence that funding has increased although one positive sign is a year-on-year increase in published articles on HPSR-related topics (Adam *et al.*, 2011; English and Pourbohloul, 2017; González Block *et al.*, 2020). The flagship Global Symposium on Health Systems Research has also attracted a record number of submissions—∼3000—in 2018 from across the world, including two-thirds from authors in low- and middle-income countries (LMICs) (Macarayan *et al.*, 2019). However, the most recent analysis of aid funding for global health revealed that <2% of all global health activity is allocated to HPSR (Grépin *et al.*, 2017).

Has this situation begun to change? How did the coronavirus disease 2019 (Covid-19) pandemic impact investment in HPSR? These are difficult questions to answer for several reasons. The latest available pieces of evidence on funding flows of HPSR financing are incomplete, tend to focus on donor commitments (which differ from real allocations) and are several years out of date. Furthermore, these financial estimates lack crucial detail on the content of HPSR and its integration within global health projects, which crude figures cannot capture. In other words, a global health project may, on the books, appear to fall within HPSR but in practice fail to meet HPSR aims and objectives, and—conversely—a project might not appear relevant to HPSR but have elements within it that do fit the HPSR definition.

If global health funders clearly disclose HPSR investments in budget lines in their annual reports or financial statements, that would open up the possibility to systematically collect and analyse relevant information. However, this is not the case; our exploratory search on the websites and financial reports of the Bill & Melinda Gates Foundation, the Global Fund, the World Bank and the United States Agency for International Development (USAID) did not yield such information (USAID, 2021; Bill & Melinda Gates Foundation, 2022; The Global Fund, 2022a; World Bank, 2022a). Indeed, data on project spending were highly aggregated, making it impossible to differentiate whether and how HPSR components were included in different spending areas (McCoy *et al.*, 2009b; Grépin *et al.*, 2017; Dieleman *et al.*, 2019).

To our knowledge, there is no systematic surveillance or real-time tracking of HPSR. Although the Alliance for Health Policy and Systems Research of the World Health Organization (WHO) produces reports on fund flows, its coverage of HPSR allocations is inevitably incomplete, as it reflects an underlying lack of fine-grained data. To address this gap, in this article, we develop a novel approach to identify HPSR in global health. We conducted a comprehensive search of calls for funding published between 2015 and 2021 on Devex.com, the world’s largest database of funding opportunities related to international development. It offers the most reliable and up-to-date coverage of actual planned HPSR in real time. It can generate new information on HPSR donors, their priorities and the scope and scale of HPSR projects. As far as we are aware, this is the first time that this data source has been used for this purpose. However, this data source is not without limitations (e.g. not all funders upload their funding calls on the platform), so it is best used in conjunction with other established approaches in order to generate a more complete picture of the HPSR landscape.

## Methods

### Search strategy

To identify HPSR funding opportunities, we performed a systematic search of the Devex.com database for funding calls that were published between 2015 and 2021. Devex.com is a leading website for professionals working in development (including global health) and maintains an extensive list of funding and consulting opportunities. Even so, Devex.com data are best seen as a large sample of data on HPSR opportunities even if we cannot claim that they are representative of the universe of such funding opportunities and there is likely some selection bias, as not all funders upload tenders on the platform. For this reason, caution in interpreting the results is necessary: the analysis of funding trends on Devex.com reveals information on international procurement for HPSR services by major global health funders, especially vis-à-vis short-term consulting opportunities.

For the purposes of our funding search, we operationalize HPSR using the following terms: research AND (‘health policy’ OR ‘health system’ OR ‘health systems’). By relying on these terms, we have taken a conservative approach, as there is a possibility that we do not capture potentially relevant funding opportunities that do not use any of these terms. Even so, given our ambition to capture funder interest in HPSR, we consider the current approach defensible in the absence of any obvious alternative.

**Figure 1.**
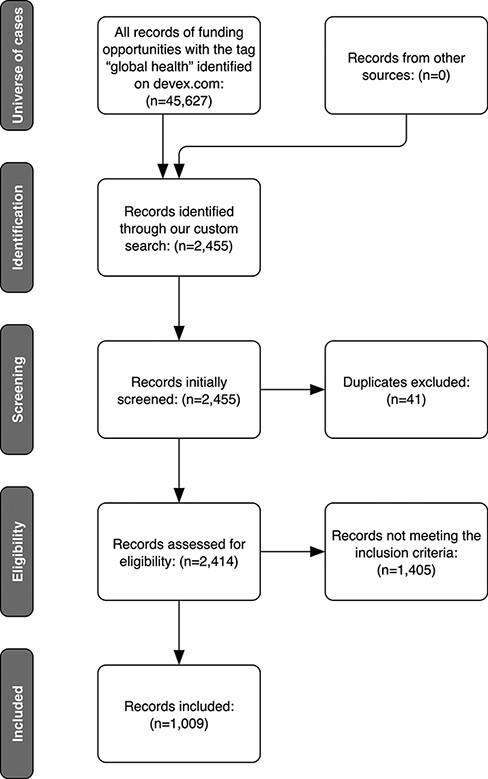
The flowchart of funding search on Devex.com for the 2015–21 period

Our search included opportunities appearing in all Devex.com funding categories (‘programs’, ‘tenders & grants’ and ‘contract awards’) and had a call status as ‘open’, ‘closed’ or ‘forecast’. Figure 1 shows our inclusion flowchart. As summarized in the figure, our initial search yielded 2455 listings, which we manually extracted.

### Selection criteria

Funding opportunities were considered for inclusion if they related specifically to HPSR funding or if there was an HPSR component in the funding remit. We excluded other types of funding (e.g. biomedical or solely related to an intervention without a clear HPSR component).

To define HPSR for inclusion, we drew on the WHO definition of HPSR as ‘the production of new knowledge to improve how societies organize themselves to achieve health goals’ (Alliance for Health Policy and Systems Research, WHO, 2007). This includes generating reliable evidence on health policy and systems, strengthening domestic capacities to undertake such research and promoting its application in health interventions (Ghaffar *et al.*, 2017). This yielded the following inclusion/exclusion criteria: (1) relevance to HPSR (basic research projects on health policy, projects targeting health system reforms that include evidence collection and/or analysis, grants to strengthen domestic research capacities or tenders to pilot and study new health policy interventions, but excluding clinical trials and health interventions without a research component); (2) referring to LMICs or specifying the funding scope as regional or worldwide; (3) having appeared on Devex.com with deadlines between 1 January 2015 and 31 December 2021 (this includes a limited number of calls that were published earlier but had 2015 closing dates) and (4) published in English.

We screened the 2455 initially identified funding calls, based on title and description. We identified 1009 as eligible. Projects with inadequate information to judge the scope of the project and HPSR relevance were excluded.

### Data extraction

For each of the 1009 funding opportunities, we extracted the following information and inputted it into an Excel file (Supplementary Appendix 1): title of project, short description of the objectives of the project, funder, recipient country, opportunity size per Devex.com classification (small—<$1 million; medium—$1–5 million; large—>$5 million) and exact values were used where available. (Most funding calls did not state target amounts. Where reported, values were commonly stated in USD, and we converted any other currencies into USD based on the average exchange rate of the year of the closing date for the call.) We retrieved all information possible from Devex.com to better understand what kinds of funding calls were being published that included an HPSR component.

Our account proceeds in two steps. First, we present the broad trends in HPSR financing by generating quantitative evidence from our search. Subsequently, we delve deeper into a qualitative elaboration of the remit and focus of our eligible funding opportunities.

## Results

### Trends in HPSR financing

**Figure 2.**
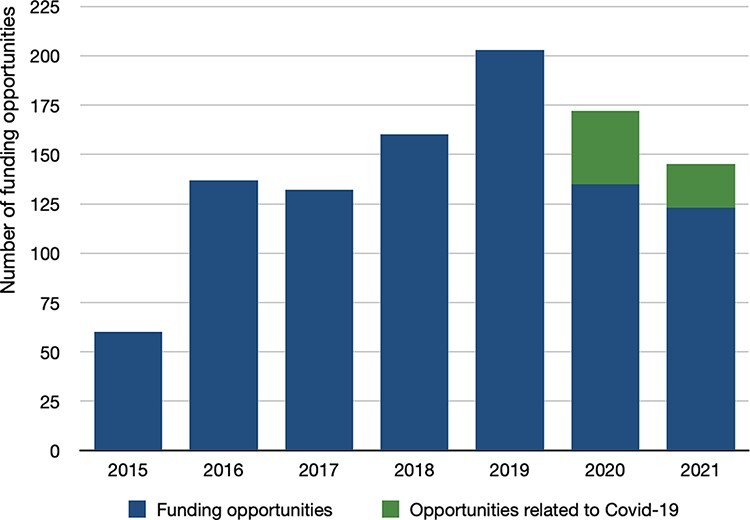
Funding opportunities on Devex.com, 2015–21

Note: Opportunities related to Covid-19 refer to any funding call that references Covid-19 in the title or project objectives.

Between 2015 and 2021, 45 627 funding calls appeared on Devex.com within the global health funding area. Of these, 1009 met our eligibility criteria—i.e. only 2.2% of all calls within Devex.com during the period of investigation. This figure parallels findings by Grépin *et al.* (2017) showing that HPSR ‘only represents approximately 2% of all donor funding for health and population projects’ (but note that our data are on funding calls, while the study by Grépin *et al*. is on amounts committed). As shown in Figure 2, over the 2015–21 period, funding opportunities appearing on Devex.com have numbered on average 144 per annum. In the period since the onset of the Covid-19 pandemic, the total number of advertised funding opportunities declined between 2020 and 2021. During this period, 19% of funding calls had a—direct or indirect—link to Covid-19. This suggests an even steeper drop in HPSR opportunities that are unrelated to Covid-19.

Who are the major funders of HPSR? As shown in Table 1, the country that is the source of the largest contributions is the USA, accounting for a third of all opportunities posted on Devex.com. The US government is the world’s largest bilateral funder of health sector interventions in dollar terms (McCoy *et al.*, 2009a), and its prominence in our data is not surprising: it reflects the sheer size of its aid budget, rather than necessarily a more concerted interest in HPSR compared with other donors. The USA’s funding for HPSR was provided through its various agencies with a remit on global health, like the National Institutes of Health, the Centres for Disease Control and the US Agency for International Development. Second, international organizations collectively account for ∼40% of all HPSR funding calls, most commonly the WHO and other United Nations (UN) specialized agencies (notably, the United Nations Children's Fund (UNICEF) and the United Nations Development Programme). The World Bank Group also stands out, advertising 9.2% of calls in our data. Surprisingly, even though the Bill & Melinda Gates Foundation is a major global health funder, including for HPSR (Grépin *et al.*, 2017), it does not feature prominently in our data, accounting for just 3.5% of entries. This is likely because the organization normally takes a proactive approach to identifying potential grantees, rather than relying on public calls (Bill & Melinda Gates Foundation, 2010). Finally, ∼5% of funding opportunities reflect multi-funder partnerships, often collaborations between international organizations and bilateral funders.

**Table 1.** Key HPSR funders

Funder type	Funder	Frequency	Share
International organizations	WHO	141	14.0
	UN system organizations (excl. WHO)	118	11.7
	Multilateral development banks	108	10.7
	Non-UN international organizations	42	4.2
Bilateral funders	USA (through agencies like CDC, the National Institutes of Healthand USAID)	335	33.2
	UK	46	4.6
	All other bilateral funders	52	5.2
Foundations	Bill & Melinda Gates Foundation	35	3.5
	All other foundations	31	3.1
Collaborations between multiple organizations		49	4.9
Other funders (non-governmental organizations, private sectors and LMIC governments)		52	5.2
**Total**		1009	100

**Figure 3.**
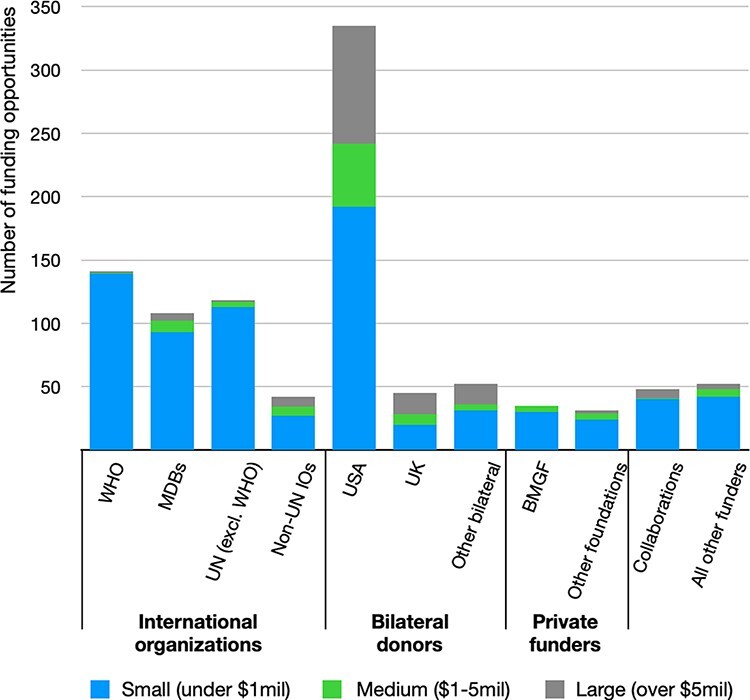
Funding opportunities on Devex.com, by funder and size

The funding calls we identified ranged from small-scale projects to multimillion-dollar opportunities. Grants of <$1 million make up nearly three-quarters of all entries in our data, those in the $1–5 million range comprise 10% and 15% of calls had a budget exceeding $5 million. As shown in Figure 3, most funders—excluding bilateral ones—advertise primarily small opportunities. In line with the increasing reliance on consultants by international organizations (Seabrooke and Sending, 2020), these smaller calls commonly seek consulting services, where individuals or companies are expected to submit competitive offers to conduct HPSR. Medium and large opportunities were primarily advertised by bilateral funders, with the US Department of Health and Human Services, of which the National Institutes of Health and the Centers for Disease Control and Prevention (CDC) are a part, standing out as the provider of 43% of all opportunities with a budget exceeding $1 million. We elaborate on the key characteristics of these larger projects in the subsequent section.

The HPSR funding calls identified in our dataset were related to diverse countries or regions, as shown in Table 2. In particular, 46% pertained to projects with a worldwide or regional scope. These projects often asked for large cross-national analyses or for evidence collection to support the development of regional health policy instruments: 27% of these projects were worth >$1 million and accounted for approximately half of the larger funding opportunities in our data. In contrast, country-specific opportunities tended to be smaller-sized projects, like calls for consultancies to collect or analyse data pertaining to a health intervention. For example, 46 of the 48 projects pertaining to the Philippines provided opportunities <$1 million, covering a wide array of health policy topics including healthy ageing, antimicrobial resistance, health accounting, hospital performance and vaccination.

**Table 2.** Top target regions/countries for HPSR financing

Scope	Frequency	Share
Worldwide scope	279	27.7
Multiple countries	92	9.1
Regional scope	88	8.7
Philippines	48	4.8
Kenya	35	3.5
India	32	3.2
Nigeria	21	2.1
Ethiopia	20	2.0
Uganda	20	2.0
South Africa	18	1.8
Bangladesh	16	1.6
Tanzania	16	1.6
Cambodia	14	1.4
Vietnam	14	1.4
Democratic Republic of Congo	13	1.3
Zimbabwe	13	1.3
Malawi	11	1.1
Zambia	11	1.1
Afghanistan	10	1.0
Mozambique	10	1.0

### Comparative evidence from funding calls

#### Grants >$1 million with HPSR components

Among large grant providers, the USA was the most prominent funder, having advertised 143 such opportunities. Many of these projects had a multi-country scope, and the HPSR aspect represented only one component among much broader projects. For example, the US CDC advertised a $45-million call to ‘conduct and monitor epidemiologic, clinical, and laboratory-based projects, surveillance, and research of important human diseases’ >5 years, with a focus on Central American countries. While much of this project was focused on epidemiological research, it also asked for research on which disease prevention and control interventions were already in place and for evidence-based proposals on how to improve policies. This included ‘operational research on public health program implementation and approaches to outbreak investigations, surveillance, and emergency preparedness’, as well as analyses of the effectiveness of different disease prevention policies (US CDC, 2018). The call did not specify what share of the budget should be directed to these ends. This project exemplifies large grants by US agencies: these were commonly opportunities with a strong epidemiological and biomedical focus but mandated that some funds would also be set aside for HPSR to underpin the translation of biomedical innovations and research findings into policy.

Similarly, other major bilateral funders pursued large-scale initiatives that included evidence-gathering and analysis components. For example, the UK Department for International Development (now merged into the Foreign, Commonwealth and Development Office) committed ∼$10 million to a project aimed at ‘Improving Mental Health in Low Income Countries’ (DFID, 2017). The key objectives included to ‘generate evidence on the implementation and scaling up of integrated packages of care for priority mental disorders in primary and maternal health care contexts, [including] generating evidence that helps to get mental health better reflected in annual operational plans at the district and national level’.

Larger grants also sought to support the development of research skills and infrastructures. For instance, the Bill & Melinda Gates Foundation committed ∼$6 million in two grants to set up a new HPSR institute in the Indian state of Bihar and ‘to support capacity building of a new cadre of Indian researchers/analysts in health systems design and to conduct relevant health systems research to contribute to improving people’s health, well-being and financial protection in India’ (Bill & Melinda Gates Foundation, 2017). In another example, a $60-million grant by the CDC in 2021 aimed to strengthen ‘public health research and implementation science (operations research) to control and eliminate infectious diseases’, with special emphasis on Covid-19. While the grant was intended primarily to support epidemiological research, it also sought to generate evidence to underpin ‘ongoing surveillance and development of COVID-19 mitigation strategies, including vaccination, and will contribute to the evidence base needed to guide interventions and enhance public health policy in [the target countries]’ (US CDC, 2020). This is an example of an HPSR strengthening project that is embedded in larger-scale biomedical financing.

The larger grants provided by multilateral development banks primarily focused on aspects of health systems that relate to financial sustainability. For example, in recent years the World Bank Group has advertised projects on expenditure tracking, health information systems, monitoring and evaluation of health system performance and operational service delivery models.

#### Grants <$1 million with HPSR components

As noted earlier, small-sized funding opportunities (<$1 million) made up two-thirds of funding calls in our data. The largest funder for such projects was again the USA (192 calls), followed by the WHO (139 calls) and other UN system organizations (113 calls). Most of these calls were invitations for the provision of consulting services to supplement funders’ ongoing projects. For example, between 2018 and 2021, the WHO advertised seven small-scale calls to support its activities in Fiji. These included wide-ranging projects such as consultancies on HIV prevention, training for local officials, the development of policy recommendations for cervical cancer elimination and the piloting and evaluation of a telemedicine solution for diabetes care. More generally, for the WHO and other UN system organizations, most funding opportunities related to specific countries (as opposed to multi-country, regional or worldwide projects) and targeted health system strengthening with an HPSR component, like data collection, performing a study or capacity building.

This small project category also includes 78% of all Covid-19-related funding calls. These were mostly consulting opportunities for rapid assessments and health system strengthening and were often collaborative. For instance, the WHO teamed up with the United Nations Population Fund and UNICEF to study the pandemic’s impact on maternal, newborn, child and adolescent health in Latin America, including the development of evidence-based policy analyses and recommendations. In another example, the African Union Commission and the Mastercard Foundation jointly called for health systems research on how a vaccine rollout could take place in African Union states.

## Discussion and conclusions

Our search of the Devex.com website provides, to our knowledge, the first comprehensive snapshot of open and internationally competitive calls for HPSR projects. These represent a subset of HPSR funding opportunities available worldwide but nonetheless offer insight into the priorities of major global health funders. Overall, our search shows a relative neglect of HPSR, as only *∼*2% of all funding calls included a discernible HPSR component. Despite increases in funding calls until 2019, this situation reversed in 2020, likely reflecting the redirection of resources to rapid assessments of the impacts of the Covid-19 pandemic. Furthermore, most of the identified projects represented small-scale opportunities—commonly for consultancies or technical assistance. To the extent that new data were generated, it either was tied to a specific large intervention or was narrow in scope in order to meet a specific challenge—often the latter type of data generation underpinned the development of policy responses to the Covid-19 pandemic.

Our analysis has important limitations. First and most notably, Devex.com is not an exhaustive list of funding opportunities. Some major funders, like the French or Japanese development agencies, or the Global Fund and the Vaccine Alliance do not appear prominently, reflecting organizational choices not to advertise on Devex.com and instead use their own procurement and advertising policies. In addition, HPSR funding opportunities provided by LMIC governments are very rare (only three in our dataset), likely due to HPSR projects being advertised through domestic channels rather than internationally. Judging from the very high frequency of small-scale funding opportunities on the website, it is possible that funders primarily rely on this website to advertise limited-scope projects—e.g. for consultants or technical assistance.

Second, detailed information was occasionally missing from the projects that we identified. Most commonly, missing data pertained to the amount of funding available, even though the opportunity was still classified according to the small/medium/large classification. A very limited set of funding opportunities only provided very short and general information on the tasks to be undertaken. In those cases, we erred on the side of exclusion from our study, thereby possibly undercounting the number of projects with an HPSR component. Our attempt to achieve greater clarity by searching the web pages of major funders for HPSR-focused projects was unsuccessful, but subsequent research can pursue this task (potentially by using machine learning techniques to conduct quantitative text analysis on text scraped from funders’ websites, where that is legally permissible).

Third, it was not possible to examine the characteristics of beneficiaries, including their geographic distribution. A focus on a particular LMIC does not mean that the funds will be directed to researchers, consultants or consortia within that country, as funding calls are often open to bidders either from around the world or only from the country of the donor. Such information is not directly reported by Devex.com, but it could be identified through consulting individual tender documents. This empirical exercise is beyond the scope of the current article, but future studies can delve deeper into the characteristics of financing arrangements of major funders.

Notwithstanding these limitations, Devex.com can provide an important and insightful snapshot of HPSR activities in real time. Indeed, given the haphazard reporting by major funders and the common delays in data publication, the approach presented here can help scholars and practitioners spot emerging priorities in global health financing and HPSR, as well as areas of persistent neglect. Furthermore, it can aid global policy efforts—including by the WHO—by providing evidence that will help strengthen efforts to increase the prominence of HPSR and institutionalize its inclusion in funding calls by major donors.

Our findings provide further depth to ongoing policy discussions on global health. A series of reports, in particular those of the Independent Panel for Pandemic Preparedness & Response (2021) and the Pan-European Commission on Health and Sustainable Development (WHO, 2021), have highlighted how underinvestment in health systems contributed to the lack of preparedness for the COVID-19 pandemic. The International Monetary Fund, whose earlier policies have been linked to weakened health systems (Kentikelenis *et al.*, 2015; Kentikelenis, 2017; Stubbs *et al.*, 2017; Forster *et al.*, 2020), has published a report calling for them to be strengthened (Sands *et al.*, 2022). The seventh replenishment of the Global Fund against acquired immune deficiency syndrome, tuberculosis and malaria has exceeded some expectations, even if still falling short of the target (The Global Fund, 2022b). A new Financial Intermediary Fund for Pandemic Prevention, Preparedness and Response has been established under the auspices of the World Bank (World Bank, 2022b). A UN high-level meeting on pandemic preparedness is planned for 2023 (Heilprin, 2022). While it is important to avoid complacency, there are some grounds for optimism that investment in health will remain high as a priority. Yet this investment will only be effective and sustainable if it becomes embedded in health systems, which in turn will only happen if it takes full account of the local context.

It is here where HPSR can make a crucial contribution. The history of development assistance is littered with examples of ideas that were implemented without considering the context, whether this was the local geography or climate, the supporting infrastructure, the institutional and governance structures and processes or the beliefs and preferences of those involved. In contrast, there is now good evidence of how HPSR can address these issues (Strachan *et al.*, 2022). Yet the capacity to undertake such research is limited in large parts of the world (Tangcharoensathien *et al.*, 2022). The challenges involved should not, however, be underestimated. As we have discussed earlier, it is often difficult to determine what should be considered as HPSR, with research that can inform the implementation of health policies arising from many different disciplines and subject areas. However, pragmatic measures to address this problem have been proposed (Mirzoev *et al.*, 2022), highlighting the importance of institution building and strengthening. This process would be facilitated by having a better source of data on existing activity than Devex.com, although again the challenges involved, in a world where organizations too often compete when the public interest would be better served by collaboration, should not be underestimated.

## Supplementary Material

czac109_Supp

## Reflexivity statement

This manuscript deals with a global issue pertaining to LMICs: global donors’ financing for HPSR. The study was undertaken jointly by academics and practitioners at the Alliance for Health Policy and Systems Research, WHO, thereby ensuring diverse inputs into the manuscript by appropriate stakeholders (the WHO has the leading role in tracking the issues covered in the manuscript). Furthermore, the manuscript is co-authored by scholars at different levels of seniority: L.D.Z. holds an MA degree and is a WHO technical officer, A.K. is a mid-career academic, A.G. is a senior WHO official, and M.M. and D.S. are senior academics.
